# Optimization of Shapes and Sizes of Moth-Eye-Inspired Structures for the Enhancement of Their Antireflective Properties

**DOI:** 10.3390/polym12020296

**Published:** 2020-02-02

**Authors:** Ji Seong Choi, Joon Hyung An, Jong-Kwon Lee, Ji Yun Lee, Seong Min Kang

**Affiliations:** 1 Department of Mechanical Engineering, Chungnam National University, Daejeon 34134, Korea; djaakzzdj@naver.com (J.S.C.); oro1yan@naver.com (J.H.A.); 2 National NANOFAB center, Division of Nano-Convergence Material Development, Daejeon 34141, Korea; jklee7@nnfc.re.kr (J.-K.L.); jylee@nnfc.re.kr (J.Y.L.)

**Keywords:** moth-eye structures, inverse-moth-eye structures, anti-reflective surfaces, diffraction grating effect, double replication method

## Abstract

Novel antireflective (AR) structures have attracted tremendous attention and been used in various applications such as solar cells, displays, wearable devices, and others. They have also stimulated the development of several other methods, including moth-eye-inspired technologies. However, the analyses of the shapes and sizes of nanostructures remain a critical issue and need to be considered in the design of effective AR surfaces. Herein, moth-eye and inverse-moth-eye patterned polyurethane-acrylate (PUA) structures (MPS and IMPS) with three different sizes are analyzed and compared to optimize the designed nanostructures to achieve the best optical properties pertaining to maximum transmittance and minimum reflectance. We fabricated moth-eye-inspired conical structures with three different sizes using a simple and robust fabrication method. Furthermore, the fabricated surfaces of the MPS and IMPS structures were analyzed based on the experimental and theoretical variation influences of their optical properties according to their sizes and shapes. As a result of these analyses, we herein propose a standard methodology based on the optimal structure of IMPS structure with a 300 nm diameter.

## 1. Introduction

Numerous studies have been conducted in the effort to develop a novel antireflective (AR) surface. There are many ways used to synthesize the AR surface [1,2,3,4], but nature-inspired structures with moth-eye-shapes are typically applied to a wide range of applications [5,6,7,8,9,10]. In particular, bioinspired surfaces are applied to optical devices such as in the display panels of electronic devices [11,12], and to eco-friendly energy devices such as solar cells [13,14,15,16]. These AR surfaces of moth-eye-inspired structures have various shapes and sizes depending on the fabrication method, thus realizing increased optical performances. For instance, processes such as direct etching [17,18,19], sol-gel [20,21], soft-lithography, and nanoimprinting processes have been used [22,23]. However, the effectiveness of different shapes is revealed in the optical properties of the structure. The most important point of the AR surface is that the fabricated tiny structures on the surface demonstrate gradual changes of the refractive index on the interface [24]. In other words, when light enters a substance with a high-refractive index from air with a refractive index value of 1, there are a lot of reflections on the interface of the surface. Therefore, to reduce the variation of the refractive index between the interfaces, the fabricated nanostructure on the surface is smaller compared to the wavelength of the incident light. In this manner, many studies focused on the materials [25,26,27,28] based on which AR surfaces are composed of and their structures [29,30,31]. However, the analysis of the shape and size of nanostructures has to be considered for designing effective AR surfaces.

In this work, moth-eye and inverse-moth-eye patterned polyurethane-acrylate (PUA) structures (MPS and IMPS) with the sizes of 300, 500, and 1000 nm are compared and analyzed to optimize the nanostructures to achieve the best optical transmittance and reflectance properties. First, we utilized a photolithography process to fabricate a moth-eye-inspired silicon master mold, and sequentially replicated IMPS samples possessing conical shapes with sizes of 300, 500, and 1000 nm from the mold. In addition, the double replicating process was used with the same polymer material for the fabrication of MPS from the prepared IMPS sample. We then analyzed the fabricated surfaces with a spectrum analyzer, showing that they vary in terms of their optical properties according to the size and shape of the structure. Additionally, the refractive index profile is shown such that there is a theoretical difference between the MPS and IMPS. As a result, we propose one of the standard methods to optimize the size and shape of the moth-eye structures based on the analysis of the experimental and theoretical results to develop surfaces with increased AR properties.

## 2. Materials and Methods

### 2.1. Preparation of 300, 500, and 1000 nm Moth-Eye Silicon Masters

Prior to the fabrication of the moth-eye silicon master, an 8 in silicon wafer (LG Siltron, Gumi, Korea) was precleaned with SC-1 solution composed of DI water, NH_4_OH, and H_2_O_2_. To fabricate dorm patterns with the three chosen sizes (300, 500, and 1000 nm) for the three moth-eye structures, a photoresist (LX-429, Dongjin Semichem, Seoul, Korea) layer with a thickness of 1000 nm was spin-coated over the precleaned wafer. On top of the surface, a pillar array with sizes of 170, 250, and 600 nm, respectively, was constructed with hexagonal shapes using a photolithographic process. The prepared wafers were then etched at the depths of 180, 300, and 500 nm with anisotropic methods using Cl_2_ and HBr gas plasma in the inductive coupled plasma (ICP) system. After removal of the PR layer and the flattening of the top shape of the pillar arrays, the surfaces of wafers were thermally oxidized with gas flows of H_2_ (4 slm) and O_2_ (12 slm) at atmospheric pressure to develop thin SiO_2_ layers with thicknesses equal to 100, 300, and 330 nm on the surface of the preconstructed basic moth-eye structure. Finally, the nitride deposition (with a thickness of 10 nm) was achieved with the low-pressure chemical vapor deposition (LPCVD) process to successfully fabricate moth-eye dorm structures with sizes equal to 300, 500, and 1000 nm.

### 2.2. Fabrication of PUA Moth-Eye Structures

First, to fabricate the moth-eye-patterned PUA structure (Changsung Sheet) (MPS), octafluorocyclobutane (C_4_F_8_) gas was deposited on the prepared silicon master surface to facilitate the replication process. After the PUA was spread over the silicon master, a substrate of polyester (PET) film was slightly contacted with it. Then, the surface was exposed to UV irradiation with 40 W (λ = 310~400 nm, Fusion Cure System, Minuta Technology, Osan, Korea) for 30 s to cure the PUA resin. Subsequently, the inversed MPS (IMPS) was obtained by separating the hardened PUA surface from the silicon master. In addition, the same replication process using other IMPS as a second mold was carried out to obtain MPS, which is a double replication method.

### 2.3. Characterization

The transmittance and reflectance were measured by an ultraviolet-visible (UV-Vis) spectrum analyzer (LAMDA 365, Scinco Corporation, Seoul, Korea) within the wavelength range of 300 nm to 800 nm. The spectrum analyzer was equipped with an integration tool to carry out the analysis of straight and diffused reflections. In addition, images were acquired by a field emission scanning electron microscopy scanner (FE-SEM, Hitachi High-Technologies Corporation, Tokyo, Japan) to observe the morphologies of MPS and IMPS.

## 3. Results and Discussion

### 3.1. Fabrication of Antireflective Moth-Eye Patterned Surfaces

**Figure 1:**
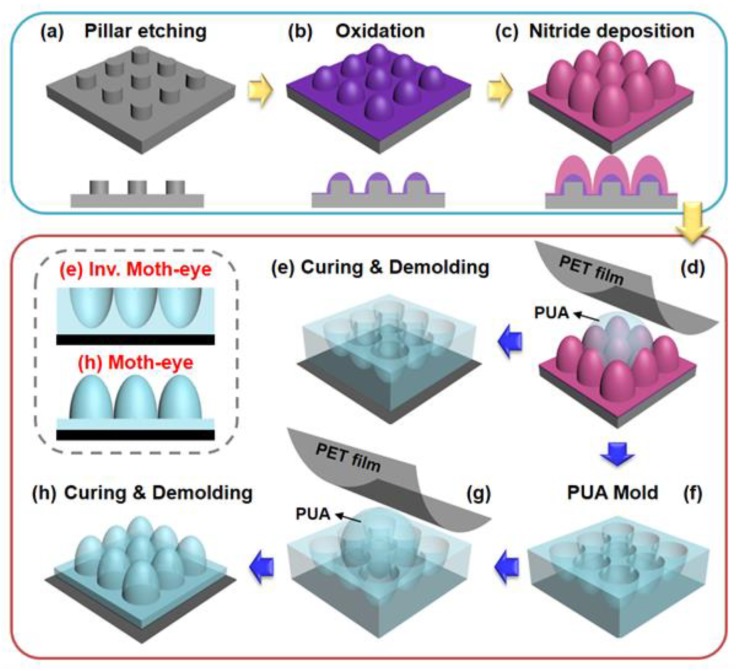
Schematics of silicon master (blue box) and MPS/IMPS fabrication processes (red box). (**a**) Cylindrical, regular pillar fabrication using photolithography and reactive ion etching on a silicon wafer surface; (**b**) Transformed cone-shaped oxidizing pillar on the silicon surface based on thermal oxidation; (**c**) Nitride deposition with the use of LPCVD to increase the height of the moth-eye structures; (**d**–**h**) PUA replication process using soft lithography based on the prepared silicon master mold. Isometric (**h**: curing and demolding) and cross-sectional shape (**e**,**f**: Inverse MPS and MPS) of IMPS (**e**) and MPS (**h**), respectively.

**Figure 2:**
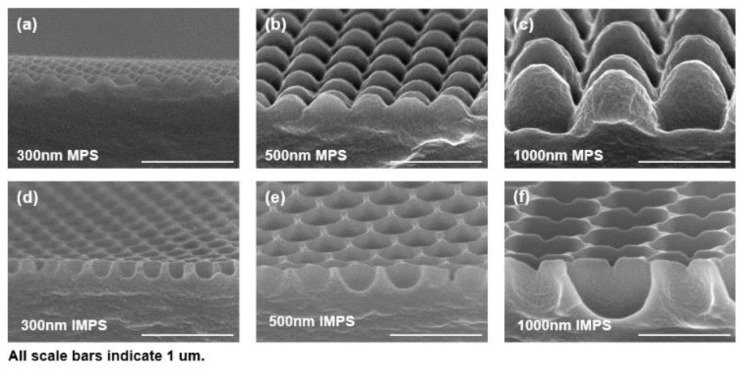
(**a**–**c**) SEM images of MPS arrays with three different sizes (300, 500 and 1000 nm) composed of PUA after the use of the double replication method; (**d**–**f**) SEM images of IMPS arrays in the same order as that depicted in (**a**–**c**).

Figure 1 shows the entire fabrication process of the moth-eye and inverse-moth-eye patterned PUA structures (MPS and IMPS) based on the use of soft lithography and a double replication method from a silicon master. Figure 1a–c shows the preparation of a silicon master at three different dimensions (300, 500, and 1000 nm) using a conventional microelectromechanical system (MEMS) process. Each regular column shape (sizes of 170, 250, and 600 nm) is fabricated on a precleaned silicon wafer based on the utilization of photolithography and reactive ion etching (RIE), as shown in Figure 1a. Next, the surface is oxidized by a thermal oxidation method that changes the column pillar form to the conicoid features of the moth-eye structure with SiO_2_ (Figure 2b). A silicon nitride layer was deposited on the surface with the LPCVD process (Figure 2c). Finally, three different silicon masters were fabricated with different moth-eye structures (300, 500, and 1000 nm).

Figure 1d–h indicates the replication of the MPS and IMPS composed of PUA. First, an octafluorocyclobutane (C_4_F_8_) gas layer was deposited on the silicon master to facilitate a soft lithographic and replication process by lowering the surface energy of the silicon master mold. After a certain amount of PUA prepolymer was spread on the silicon master mold, it was covered and lightly pressed with a PET film which was used as a substrate (Figure 1d). The PUA prepolymer, which was placed between the PET film and silicon mold was exposed to UV rays and cured for approximately 15 s (blue arrow). The IMPS surface (Figure 1e) was then obtained based on the gentle separation of the cured PUA film from the silicon mold. The cross-sectional shape of the IMPS is shown in Figure 1e and highlighted with red bold text. Figure 1f,g shows that the MPS is fabricated based on the same process using other IMPS samples as the mold, which constitutes the double replication method. Finally, we successfully fabricated both the MPS (Figure 1h) and IMPS (Figure 1e) surfaces with simple and robust replication methods.

### 3.2. Specific Shapes of MPS and IMPS Based on SEM Analyses

Figure 2 shows the results of SEM analyses generated to examine the specific shapes of the fabricated MPS and IMPS. As shown in the cross-sectional SEM images, although the final structural height of all the samples looks lower than that of the prepared silicon master owing to the tilting angle in the SEM measurement process, it has been demonstrated that the morphologies of MPS and IMPS have high-structural fidelities. Figure 2a–c show the SEM images of the fabricated MPS (300, 500, and 1000 nm). Additionally, the IMPS of the three-dimensional structures are shown as SEM images in Figure 2d–f. The shapes of the IMPS and MPS structures are clearly observed. As a result, the MPS and IMPS, which are AR surfaces generated by the patterning of the simple PUA replicated process, were successfully fabricated in large areas.

### 3.3. Effect of Antireflective MPS

**Figure 3:**
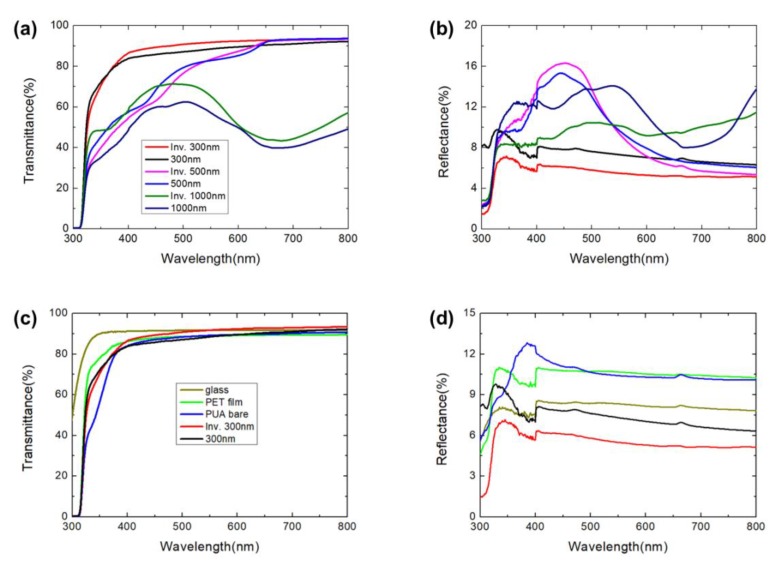
(**a**) Transmittance and (**b**) reflectance of MPS and IMPS with six different samples. Comparison of (**c**) transmittance and (**d**) reflectance with glass, PET film, PUA bare and MPS/IMPS samples with pitches of 300 nm.

We conducted transmittance and reflectance analyses to optimize the AR surface. The optical properties were analyzed with a UV-Vis analyzer. Figure 3 shows the collected optical data of the MPS and IMPS structures. The structure of the 300 nm pitch shows a very good transmittance response within the entire light spectrum. However, the surfaces with the structures of 500 nm and 1000 nm did not transmit much light at specific wavelengths, which is explained by the grating equations at the vertical light angle of incidence [24].

(1) $${\sin\theta }_{d}=\frac{m\lambda }{np},$$

In Equation (1), θ*_d_* is the diffraction angle, *m* is the order of the diffracted light, λ is the wavelength of incident light, and *n* can be expressed as *n* ≈ 1 because this value corresponds to the refractive index of air. In addition, *p* is the grating space. Accordingly, the wavelength of light generated by the dimension of the structure was confirmed based on Equation (1). Based on this equation, Figure 3a shows that the value of the measured transmittances for the 300 nm MPS and 300 nm IMPS samples were ~97% at the wavelength of 800 nm, but the values slightly decreased to ~90% at lower light wavelengths of light. The average transmittance of the 300 nm IMPS was 91.7% (average of 400–800 nm, visible wavelength), and had the highest value among all the measured samples. This phenomenon is explained by Equation (1), which shows that light is transmitted almost completely because diffraction is not generated by outside reflections.

**Figure 4:**
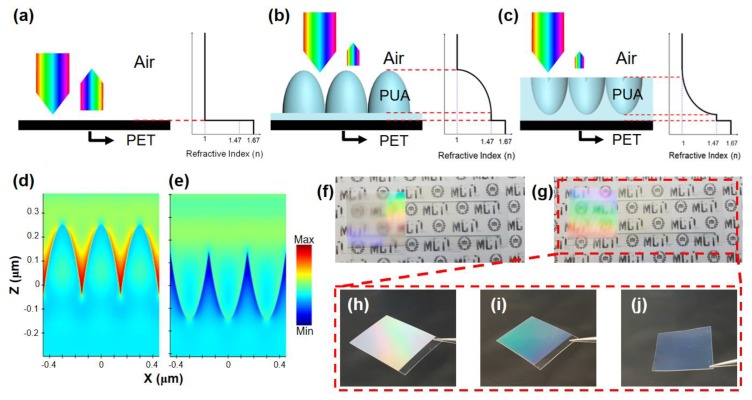
Illustrated refractive index profiles of (**a**) air/PET, (**b**) air/MPS/PET, and (**c**) air/IMPS/PET films. E-field intensity distributions of the (**d**) MPS and (**e**) IMPS at a wavelength of 520 nm. Digital camera images demonstrating structural colors for (**f**) MPS and (**g**) IMPS samples with sizes equal to 300, 500, and 1000 nm, respectively. (**h**–**j**) Magnified reflected structural colors of the three different sizes of the IMPS samples.

The 500 nm MPS and IMPS samples also confirmed very high transmittance at the wavelength of 800 nm. However, the intensities of the measured transmittance profiles of the 500 nm samples decreased abruptly from 600 nm by the first-order external reflection owing to the diffraction effect, as described by Equation (1). Additionally, MPS and IMPS structures (1000 nm) demonstrated lower transmittance values, which were approximately 50%, owing to the effects of several orders of diffractions (up to fourth order). This is shown by the iridescent surface depicted in Figure 4f.

Figure 3b shows that the collected data represent the reflectances of the fabricated MPS and IMPS structures with three different dimensions. First, the results exhibit low average reflectance values, whereby the MPS and IMPS samples (300 nm) were approximately equal to 7% and 5.4%, respectively. Given that the transmittance performances were similar, the reflectance performances of the low-wavelength range gradually increased, while the reflectance responses near 350 nm had increased intensities owing to the grating effect. Based on the same principle, the MPS and IMPS structures (500 and 1000 nm) yielded increased reflectances of approximately 9% or more. Thus, the experimental results show the outstanding optical properties of the 300 nm surface without grating diffraction. Interestingly, Figure 3a–b demonstrates that all the IMPS sizes have higher transmittance and lower reflectance values than the respective sizes of the MPS samples.

To investigate the superior optical properties of the fabricated samples with 300 nm structures, the transmittance and reflectance of the MPS and IMPS structures (dimensions of 300 nm) were compared with commonly used glass, a substrate PET film and base materials of PUA bare, as shown in Figure 3c–d. The average transmittances of glass, the PET film and PUA bare with wavelengths in the range of 400–800 nm were approximately 91.7%, 88.8% and 89.1%, respectively. However, the corresponding transmittances of the MPS and IMPS structures were 89% and 91.7%, respectively, even though the refractive index of the PUA was larger than that of glass. The transmittance of MPS was larger than that of glass for light wavelengths over 700 nm. The optical value of IMPS is higher (at the wavelength of 550 nm) than those of glass (wavelengths >400 nm), the PET film, and PUA bare, as shown in Figure 3c. Both the MPS and IMPS have better transmittance values compared to the substrate PET film and PUA bare. Additionally, the reflectances of MPS and IMPS were approximately 7.4% and 5.5%, respectively. Both values are lower than those of glass, the PET film and PUA bare, which are 8%, 10% and 10.5%, respectively. Despite the use of high-refractive index materials (n_PET_ ~ 1.67 and n_PUA_ ~ 1.46), MPS and (especially) IMPS improved their optical properties.

### 3.4. Structural Analysis of MPS to Prevent Reflections

As a result of the transmittance and reflectance analyses with the use of several samples, it was confirmed that the inverse shape of the moth-eye structure had the best optical properties, as previously shown in Figure 3a,b. The reason for these phenomena is schematically explained in Figure 4a–c. When an incident light passes through various substances, sudden changes of the refractive index lead to an increased number of light reflections at the surface interface. In Figure 4a, increased reflectance occurs because the refractive index changes rapidly when light reaches the PET film (n ~ 1.67) from the air medium (n ~ 1). However, nanostructures caused the refractive index to gradual change by the structural effect of the moth-eye shape as shown in Figure 4b,c. As a result, both the fabricated MPS and IMPS structures have small reflectances owing to the reduction of the reflectance achieved by the gradual decrease of the refractive index, as illustrated in the schematics. Moreover, the refractive index profile derived from the structure’s shape constitutes the reason for which the optical properties of IMPS are better than those of the MPS, as previously analyzed in Figure 3. The MPS outcomes show that the value of the refractive index profile was rapidly increased in a similar manner to the precedent staircase graph of the PET film. By contrast, IMPS outcomes show that its value changes gradually and slowly, as indicated by the refractive index graph of Figure 4b,c. Therefore, it is shown that the IMPS with the smoothly and gradually changing refractive index had better transmittance and reflectance responses. This phenomenon is proven by simple simulation result in Figure 4d,e, demonstrating that the E-field intensity of IMPS is relatively weaker than that of the MPS due to its superior AR property. Based on this analysis, the optical properties of both the MPS and IMPS were verified by the digital camera images of Figure 4f–j. Figure 4f shows the MPS structures with sizes of 1000 nm (first from the left), 500 nm (second), and 300 nm (third). The blurred mark with the color of light dispersed and reflected at 1000, 500, and 300 nm, is transparent. Similarly, the IMPS samples demonstrate that the effects of nanostructures with the sizes of 1000 nm, 500 nm, and 300 nm, are the same as those for the MPS (Figure 4g). In Figure 4h, the 1000 nm structure of the IMPS identifies the rainbow color reflecting all the wavelengths of light generated by the diffraction grating effect, as described by Equation (1). The 500 nm structure of IMPS is confirmed to be green, mainly reflecting light in wavelength bands with maximum values of 550 nm or less, while the 300 nm structure is transparent.

## 4. Conclusions

In conclusion, this study was carried out to optimize AR surfaces depending on the shapes and sizes of moth-eye structures. First, MPS and IMPS structures with three different sizes (300, 500, and 1000 nm) were fabricated with simple photolithographic and double replication processes. The replicating process used to fabricate the MPS and IMPS was conducted several times with the PUA and PET film of the substrate with the use of the prepared moth-eye-inspired silicon master mold. Specific shapes were identified based on SEM analyses to evaluate the condition of the structure. In addition, the measurements of the transmittance and reflectance were described in detail, and the properties of the moth-eye structures were explained by the diffraction grating effect for the AR surface. Finally, the shapes and sizes of the MPS and IMPS structures with the sizes of 300, 500, and 1000 nm were analyzed by a spectrum analyzer. As a result, IMPS structures with dimensions of 300 nm were proved to have more prominent optical properties based on experimental and theoretical analyses. In summary, we optimized MPS and IMPS structures as part of the standard process for AR surfaces that can increase the efficiency of optical instruments. It is envisioned that the analysis for superior bioinspired AR surfaces in this study can be used in future academic and industrial applications.
